# Toward a Conceptual Multiscale Framework for Predictive Radiobiology: Integrating Genomic Damage, Network Rewiring, and Tissue Microenvironment

**DOI:** 10.3390/ijms27125230

**Published:** 2026-06-09

**Authors:** Tae Gen Son

**Affiliations:** Research Center, Dongnam Institute of Radiological and Medical Sciences, Busan 46033, Republic of Korea; tgson@dirams.re.kr; Tel.: +82-51-720-5146

**Keywords:** predictive radiobiology, multiscale framework, network rewiring, tissue microenvironment, multi-omics, non-linear radiation response

## Abstract

Radiation-induced biological responses emerge through complex interactions across multiple biological scales, ranging from molecular damage to tissue remodeling and organism-level outcomes. Although traditional radiobiology has primarily focused on DNA damage and linear dose–response relationships, increasing evidence suggests that radiation responses are highly context-dependent and cannot be fully explained by genomic alterations alone. In particular, low-dose and chronic radiation exposures often induce biological effects that involve dynamic regulatory processes beyond direct mutational burden. The narrative review proposes a conceptual multiscale framework for predictive radiobiology that integrates genomic damage, post-transcriptional regulation, network rewiring, and tissue microenvironmental interactions. Within this framework, “predictive radiobiology” refers to the integrative prediction of radiation-induced outcomes, including radiosensitivity, tissue remodeling, fibrosis progression, therapeutic response, and long-term carcinogenic risk. We discuss how radiation-induced signaling extends beyond DNA double-strand breaks to include RNA-binding protein-mediated regulation, adaptive network responses, and extracellular matrix-dependent cellular plasticity. Recent advances in multi-omics, single-cell analysis, spatial biology, and three-dimensional organotypic models have revealed that radiation responses are governed by interconnected molecular and tissue-level processes. Furthermore, artificial intelligence and systems-level computational approaches provide new opportunities for modeling non-linear and context-dependent radiation effects across biological scales. We further discuss current limitations, including data integration challenges, reproducibility issues, and the translational gap between experimental models and clinical applications. Collectively, this conceptual framework highlights the need for integrative and multiscale approaches to improve mechanistic understanding and predictive modeling in modern radiobiology.

## 1. Introduction

Radiation-induced biological responses have traditionally been interpreted through a DNA-centric framework, in which double-strand breaks (DSBs) are considered the primary determinants of cellular fate [1,2,3]. This paradigm has led to the development of linear dose–response models that assume a proportional relationship between radiation dose and biological effect. While this framework has been instrumental in advancing radiotherapy and radiation protection, it does not fully capture the complexity and heterogeneity of radiation-induced biological processes. In particular, it fails to account for context-dependent responses and non-genetic regulatory mechanisms that increasingly appear to play critical roles in determining cellular outcomes.

Accumulating evidence indicates that radiation responses are governed by interactions across multiple biological layers, including genomic, transcriptomic, proteomic, and signaling network levels [4,5,6]. Rather than being driven solely by DNA damage, radiation-induced effects emerge from coordinated processes that span these layers, involving both direct molecular alterations and indirect regulatory adaptations. This perspective has been supported by recent advances in multi-omics technologies, which enable the simultaneous interrogation of multiple biological dimensions and reveal complex interdependencies between them.

Moreover, radiation responses are frequently non-linear, particularly at low doses, where phenomena such as adaptive responses, hypersensitivity, and hormesis have been reported [7,8]. These observations challenge the traditional linear no-threshold (LNT) model and suggest that biological systems exhibit threshold-like or even bidirectional responses depending on dose, exposure history, and cellular context. Importantly, such non-linear behaviors cannot be adequately explained by models that rely solely on DNA damage accumulation.

In addition to molecular complexity, temporal dynamics play a critical role in shaping radiation responses. Immediate events such as DNA damage recognition, chromatin-associated signaling, and repair-factor recruitment occur within minutes to hours following irradiation, whereas downstream processes, including transcriptional and post-transcriptional reprogramming, epigenetic modulation, and network-level adaptations, may evolve over extended time scales ranging from hours to days or even longer [9,10,11]. These temporally layered responses introduce additional challenges in predicting long-term biological outcomes based solely on early molecular events.

Furthermore, the tissue microenvironment has emerged as a key determinant of radiation response. Conventional two-dimensional (2D) culture systems lack the structural, mechanical, and biochemical complexity of in vivo tissues, limiting their ability to faithfully recapitulate radiation-induced processes. In contrast, three-dimensional (3D) organotypic models and organoids provide a more physiologically relevant context by incorporating extracellular matrix (ECM) architecture, cell–cell interactions, and stromal components [12,13]. These features significantly influence radiation sensitivity, cellular plasticity, and tissue-level responses, highlighting the importance of considering spatial organization and microenvironmental cues.

Collectively, these findings indicate that radiation-induced biological effects cannot be fully understood within a single-layer framework. Instead, there is a growing need for integrative approaches that capture the multiscale nature of radiation responses, spanning molecular damage, regulatory networks, tissue microenvironmental interactions, and long-term tissue remodeling. Artificial intelligence (AI) and systems biology approaches offer powerful tools for integrating heterogeneous datasets, modeling non-linear relationships, and identifying emergent properties of complex biological systems [14,15]. These approaches are particularly well-suited for bridging the gap between descriptive and predictive radiobiology.

**Figure 1:**
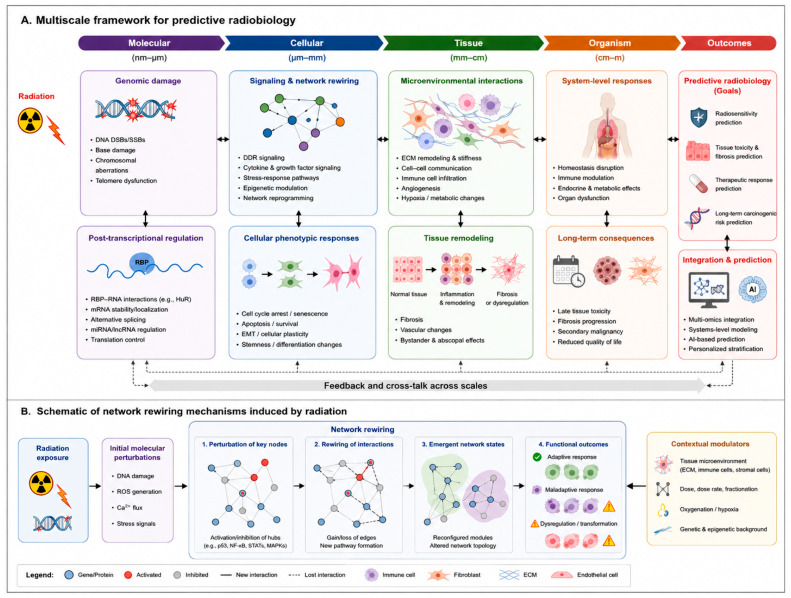
Multiscale framework and network rewiring mechanisms in predictive radiobiology. (**A**) Ionizing radiation (IR) induces biological responses across multiple organizational scales, ranging from genomic damage and post-transcriptional regulation to signaling network rewiring, tissue microenvironmental interactions, and organism-level outcomes. These interconnected processes collectively contribute to radiation-induced phenotypes, including radiosensitivity, tissue toxicity, fibrosis progression, therapeutic response, and long-term carcinogenic risk. Integration of multi-omics datasets and artificial intelligence (AI)-based computational approaches may facilitate predictive modeling across biological scales. (**B**) Schematic representation of radiation-induced network rewiring mechanisms. Initial molecular perturbations, including DNA damage, reactive oxygen species (ROS) generation, and stress signaling, dynamically reorganize regulatory networks through pathway cross-talk, feedback regulation, and context-dependent signaling interactions. These adaptive or maladaptive network states are further modulated by tissue microenvironmental factors, radiation dose conditions, and biological context, ultimately influencing long-term cellular and tissue responses. Abbreviations: AI, artificial intelligence; DDR, DNA damage response; ECM, extracellular matrix; EMT, epithelial–mesenchymal transition; LET, linear energy transfer; NHEJ, non-homologous end joining; ROS, reactive oxygen species. This figure was created and arranged by the author using Microsoft PowerPoint with manually assembled graphical elements and schematic illustrations.

In this narrative review, a conceptual multiscale framework for predictive radiobiology is presented that integrates genomic damage, post-transcriptional regulation, network-level adaptations, and tissue microenvironmental context. In this context, “predictive radiobiology” refers to integrative approaches aimed at forecasting radiation-induced outcomes, including radiosensitivity, tissue toxicity, fibrosis progression, therapeutic response, and long-term carcinogenic risk. Furthermore, this review discusses how emerging experimental platforms, including 3D organotypic models and multi-omics approaches, together with AI-based computational modeling, may improve the mechanistic understanding and prediction of radiation-induced biological outcomes across multiple biological scales (Figure 1). This narrative review was based on literature retrieved from PubMed, Web of Science, and Google Scholar. Studies published between 2000 and March 2026 were primarily considered, with particular emphasis on recent advances reported during the past decade.

This article is intended as a narrative and conceptual review rather than a systematic meta-analysis. The literature discussed in this review was identified through searches of PubMed, Web of Science, and Google Scholar using combinations of keywords related to radiobiology, DNA damage response, low-dose radiation, post-transcriptional regulation, network rewiring, tissue microenvironment, organoids, multi-omics, and predictive modeling. Rather than providing exhaustive coverage, the primary objective is to propose an integrative conceptual framework linking genomic, regulatory, network-level, and microenvironmental processes in predictive radiobiology.

## 2. Molecular-Level Responses: DNA Damage and Genomic Signatures

Ionizing radiation induces a broad spectrum of DNA lesions, including double-strand breaks (DSBs), single-strand breaks, base damage, DNA crosslinks, and complex clustered lesions [16,17]. Among these, DSBs are generally considered the most cytotoxic form of DNA damage because of their capacity to promote chromosomal rearrangements, genomic instability, and cell death if improperly repaired. Cellular responses to DSBs are coordinated through highly integrated DNA damage response (DDR) networks involving the ATM and ATR kinases, downstream checkpoint mediators such as CHK1 and CHK2, and multiple effector pathways regulating cell-cycle arrest, DNA repair, senescence, and apoptosis [18,19].

In addition to radiation dose, radiation quality and linear energy transfer (LET) substantially influence the complexity of DNA lesions and subsequent repair outcomes. High-LET radiation, including alpha particles and carbon ions, induces densely clustered and complex DNA damage that is more difficult to repair and is frequently associated with persistent genomic instability, complex chromosomal rearrangements, and altered therapeutic responses compared with low-LET radiation such as X-rays or gamma rays [20].

Two major DSB repair pathways, non-homologous end joining (NHEJ) and homologous recombination (HR), play central roles in preserving genomic integrity following radiation exposure [21]. NHEJ is a rapid but relatively error-prone repair mechanism that directly ligates broken DNA ends and is frequently associated with small insertions and deletions (indels) and structural alterations [22]. In contrast, HR utilizes homologous DNA templates to mediate high-fidelity repair but is largely restricted to the S and G2 phases of the cell cycle [23]. The balance and coordination between these repair pathways are influenced by multiple factors, including chromatin organization, cell-cycle status, radiation quality, and cellular context, thereby contributing to variability in radiation-induced genomic instability and mutational outcomes [24,25].

Recent advances in high-throughput sequencing and whole-genome analysis have enabled systematic characterization of radiation-associated mutational signatures across diverse biological systems [26,27]. These signatures encompass distinct patterns of insertions and deletions (indels), single-base substitutions, and structural variations (SVs), reflecting the underlying DNA damage and repair mechanisms activated following radiation exposure. In particular, radiation-associated indel signatures are frequently linked to error-prone repair processes, including microhomology-mediated end joining, suggesting an important role for aberrant DSB repair in shaping genomic outcomes. Structural variations, such as deletions, inversions, insertions, and translocations, further contribute to large-scale genomic reorganization and may serve as molecular footprints of prior radiation exposure.

At higher radiation doses, cells exhibit increased frequencies of complex genomic rearrangements, including chromothripsis and chromoplexy, which involve extensive chromosome fragmentation and aberrant reassembly events [28,29]. These catastrophic genomic alterations are characterized by clustered DNA breakpoints, oscillating copy-number states, and highly disordered chromosomal architectures, reflecting profound defects in DNA damage repair and genome maintenance mechanisms. The emergence of such large-scale genomic reorganization highlights the substantial impact of radiation on genome architecture and underscores the limitations of models focused solely on isolated or simple DNA lesions.

Quantitative genomic analyses have demonstrated dose-dependent increases in radiation-associated mutational burden, including insertions and deletions (indels) and structural variations (SVs), supporting a general relationship between radiation exposure and genomic instability [30,31]. However, emerging evidence indicates that this relationship is not strictly linear and may vary according to mutation class, radiation quality, and biological context [31]. Certain structural alterations, particularly complex rearrangements and clustered chromosomal abnormalities, appear to accumulate disproportionately at higher radiation doses, whereas other mutation patterns exhibit more heterogeneous or context-dependent behaviors [32,33].

In contrast to high-dose exposure, low-dose radiation frequently induces relatively modest levels of detectable genomic mutations while still eliciting substantial biological responses [34]. This apparent discrepancy suggests that genomic alterations alone are insufficient to account for the full spectrum of radiation-induced effects. Under low-dose conditions, non-genetic regulatory processes, including epigenetic modifications, chromatin remodeling, oxidative stress signaling, and adaptive cellular responses, may play increasingly important roles in shaping biological outcomes.

Beyond direct DNA damage, radiation-induced responses are strongly influenced by chromatin organization, genome topology, and nuclear architecture [35,36]. The accessibility of DNA lesions, spatial proximity between chromosomal regions, chromatin mobility, and higher-order genome organization collectively affect DNA repair pathway selection, mutational outcomes, and genomic stability [37,38]. These structural and regulatory features contribute substantially to cell-to-cell heterogeneity in radiation responses, even among cells exposed to comparable radiation doses [39].

**Figure 2:**
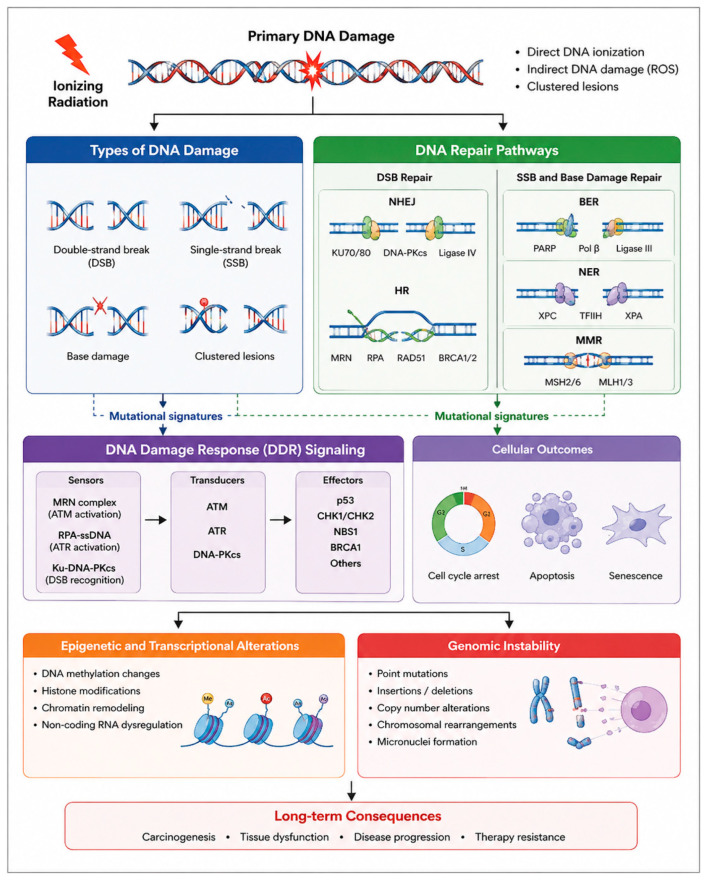
Radiation-induced DNA damage, repair pathways, and genomic instability. Ionizing radiation induces diverse forms of DNA damage, including double-strand breaks (DSBs), single-strand breaks (SSBs), base damage, and clustered DNA lesions through both direct ionization and indirect reactive oxygen species (ROS)-mediated mechanisms. These lesions activate multiple DNA repair pathways, including non-homologous end joining (NHEJ), homologous recombination (HR), base excision repair (BER), nucleotide excision repair (NER), and mismatch repair (MMR). DNA damage recognition subsequently triggers DNA damage response (DDR) signaling networks involving sensor proteins, transducers such as ATM and ATR, and downstream effectors regulating cell cycle arrest, apoptosis, senescence, and stress adaptation. Imperfect or error-prone repair processes contribute to mutational signatures, epigenetic and transcriptional alterations, and genomic instability, including insertions/deletions, chromosomal rearrangements, copy number alterations, and micronuclei formation. These molecular and genomic perturbations collectively contribute to long-term biological consequences, including carcinogenesis, tissue dysfunction, disease progression, and therapeutic resistance. Abbreviations: ATM, ataxia telangiectasia mutated; ATR, ataxia telangiectasia and Rad3-related protein; BER, base excision repair; BRCA1/2, breast cancer susceptibility proteins 1/2; DDR, DNA damage response; DNA-PKcs, DNA-dependent protein kinase catalytic subunit; DSB, double-strand break; HR, homologous recombination; MMR, mismatch repair; MRN, MRE11-RAD50-NBS1 complex; NER, nucleotide excision repair; NHEJ, non-homologous end joining; PARP, poly(ADP-ribose) polymerase; ROS, reactive oxygen species; SSB, single-strand break. This figure was created and arranged by the author using Microsoft PowerPoint with manually assembled graphical elements and schematic illustrations.

Importantly, DNA damage responses represent only the initial layer of radiation-induced biological processes. While genomic alterations provide critical insights into the mechanisms of radiation action, they must be integrated with downstream regulatory events, including post-transcriptional regulation, signaling network dynamics, and microenvironmental interactions. This perspective emphasizes that DNA damage should be viewed not as an isolated endpoint, but as a trigger for multiscale biological responses that propagate across multiple levels of organization (Figure 2).

## 3. Post-Transcriptional Regulation and Network Rewiring

Beyond genomic alterations, radiation-induced biological responses are critically shaped by post-transcriptional regulatory mechanisms that dynamically modulate gene expression independently of direct DNA sequence changes [40,41]. These adaptive processes enable rapid and context-dependent cellular responses to radiation-induced stress and may become particularly important under conditions where detectable genomic damage is relatively limited, such as low-dose or chronic radiation exposure. Regulation at the level of mRNA stability, localization, translation, and RNA-binding protein activity therefore represents an additional regulatory layer that complements transcriptional and genomic responses.

RNA-binding proteins (RBPs) play central roles in post-transcriptional regulation by coordinately controlling RNA processing, stability, localization, and translation. Among these, HuR (ELAVL1) is a well-characterized stress-responsive RBP that binds AU-rich elements within the 3′ untranslated regions (UTRs) of target mRNAs, thereby promoting transcript stabilization and enhancing translational efficiency [42,43]. HuR regulates numerous genes involved in cellular stress adaptation, inflammatory signaling, survival pathways, and immune responses, positioning it as an important mediator of adaptive and context-dependent cellular programs following stress exposure.

Recent experimental studies, including low-dose radiation-induced crosslinking immunoprecipitation (LDIR-CLIP)-based analyses, have demonstrated that radiation exposure can dynamically alter HuR–mRNA interactions in a context-dependent manner [44,45,46]. Notably, low-dose irradiation has been associated with reduced stability of specific HuR-bound transcripts, including PAX6 and NR2F6, despite relatively minimal changes in HuR protein localization [46]. These findings suggest that radiation-induced regulation of RNA stability may occur independently of canonical HuR redistribution mechanisms, revealing an additional layer of post-transcriptional adaptation under radiation-induced stress conditions.

Importantly, these findings provide a potential mechanistic framework for understanding how low-dose radiation can induce measurable biological effects despite relatively modest levels of detectable genomic mutations [47,48]. Rather than acting exclusively through cumulative DNA damage, radiation exposure may dynamically reshape RNA–protein interaction networks and selectively modulate the stability, localization, and translation of regulatory transcripts [49]. Such post-transcriptional perturbations can subsequently propagate through interconnected signaling pathways and regulatory networks, ultimately influencing broader cellular phenotypes, including inflammatory activation, stress adaptation, and cell fate determination [50].

Beyond the regulation of individual transcripts, radiation exposure can induce coordinated alterations across gene regulatory networks involving cytokines, transcription factors, signaling mediators, and stress-response pathways [51,52]. This process, often referred to as “network rewiring,” describes the dynamic reorganization of regulatory interactions and signaling architectures in response to environmental and radiation-induced stress. Rather than reflecting activation of isolated linear pathways, network rewiring represents systems-level adaptation involving feedback regulation, pathway cross-talk, and context-dependent shifts in signaling hierarchy [50,53]. For example, inflammatory signaling networks involving IL-6, IL-8, and ICAM-1 may be differentially regulated according to radiation dose, exposure duration, and tissue context, thereby influencing intercellular communication, immune modulation, and downstream tissue responses.

From a systems biology perspective, RBPs such as HuR may contribute to network rewiring by coordinately regulating functionally related groups of transcripts and integrating post-transcriptional control with broader signaling and stress-response networks [49,54]. Through these coordinated regulatory interactions, relatively subtle perturbations in RNA stability and translation may be amplified into large-scale alterations in cellular behavior, intercellular communication, and tissue-level responses [55]. Collectively, these observations support the concept that radiation-induced biological effects arise from interconnected and adaptive regulatory systems rather than from isolated molecular events alone.

**Figure 3:**
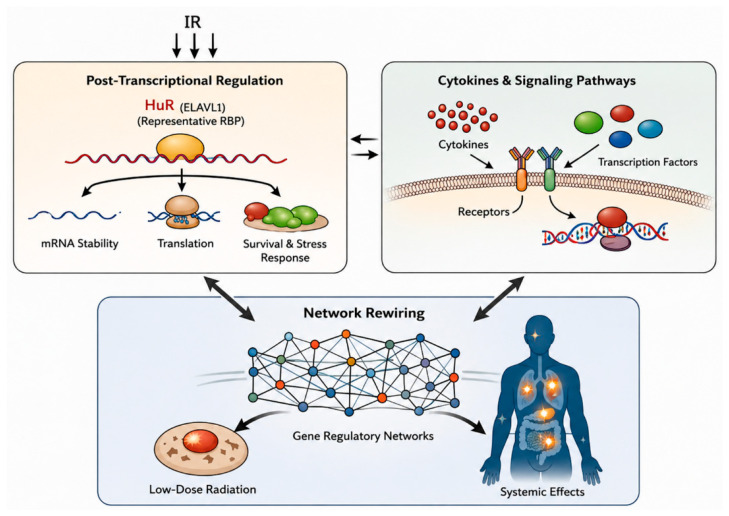
Post-transcriptional regulation and network rewiring in radiation responses. Ionizing radiation (IR) induces dynamic post-transcriptional and signaling responses that contribute to network-level adaptation and context-dependent biological outcomes. Representative RNA-binding proteins (RBPs), including human antigen R (HuR/ELAVL1), regulate mRNA stability, translation, and stress-response pathways following radiation exposure. Radiation-induced alterations in cytokine signaling and transcription factor activation further modulate intracellular communication and downstream gene expression programs. These interconnected regulatory processes collectively contribute to network rewiring through reorganization of gene regulatory networks, thereby influencing cellular adaptation, inflammatory signaling, and systemic biological responses. Under low-dose radiation conditions, relatively subtle perturbations in RNA regulation and signaling pathways may propagate through interconnected networks and contribute to broader tissue- and organism-level effects. Abbreviations: ELAVL1, embryonic lethal abnormal vision-like protein 1; HuR, human antigen R; IR, ionizing radiation; mRNA, messenger RNA; RBP, RNA-binding protein. This figure was created and arranged by the author using Microsoft PowerPoint with manually assembled graphical elements and schematic illustrations.

Collectively, these observations highlight that radiation-induced biological effects are not solely determined by genomic damage, but are also critically shaped by post-transcriptional regulation and dynamic network-level adaptations. This regulatory layer serves as an important intermediary between molecular perturbations and higher-order biological outcomes, linking genomic damage to tissue-level responses through context-dependent signaling processes. Importantly, this perspective provides a conceptual framework for understanding how low-dose radiation may exert significant biological effects despite limited detectable DNA damage, thereby extending traditional DNA-centric models of radiobiology (Figure 3).

## 4. Tissue Microenvironment and Three-Dimensional Models

The tissue microenvironment plays a fundamental role in shaping radiation responses by providing structural, biochemical, and mechanical cues that regulate cellular behavior and tissue organization [56]. Although conventional radiobiological studies have largely relied on two-dimensional (2D) culture systems, such models incompletely represent important features of native tissues, including extracellular matrix (ECM) architecture, spatial organization, mechanical heterogeneity, and multicellular interactions. As a result, simplified 2D systems may not fully capture the complexity and context dependency of radiation-induced biological processes and tissue-level responses.

Three-dimensional (3D) organotypic models and organoid systems have emerged as valuable platforms for investigating radiation responses within physiologically relevant tissue environments [57,58]. These systems reproduce important features of native tissue organization, including extracellular matrix (ECM) composition, mechanical stiffness, nutrient and oxygen gradients, and complex cell–cell interactions. Such microenvironmental characteristics actively influence signaling dynamics, cellular plasticity, stress adaptation, and tissue remodeling processes, thereby substantially affecting radiation sensitivity and downstream biological responses.

Radiation exposure in 3D systems often elicits biological responses distinct from those observed in conventional 2D cultures. Enhanced epithelial–mesenchymal transition (EMT), increased ECM remodeling, altered inflammatory signaling, and activation of pathways such as Notch signaling have been consistently reported in 3D microenvironmental contexts [59,60]. These findings indicate that radiation-induced phenotypes are strongly shaped by tissue architecture and multicellular interactions and cannot be fully recapitulated in simplified reductionist systems.

Recent studies using SVF-derived 3D organotypic models further support this concept by demonstrating pronounced EMT-like phenotypic transitions accompanied by activation of Notch signaling pathways following radiation exposure [61,62,63]. These responses are associated with increased expression of fibronectin, N-cadherin, and EMT-related transcription factors, including SNAIL, SLUG, and TWIST [62]. Notably, attenuation of these effects following γ-secretase inhibitor (GSI) treatment suggests that Notch signaling may function as an important mediator of radiation-induced phenotypic plasticity [63,64]. Together, these observations support the concept that radiation responses are dynamically regulated through context-dependent signaling interactions within the tissue microenvironment.

In addition to epithelial plasticity, stromal components of the microenvironment, particularly fibroblasts and cancer-associated fibroblasts (CAFs), play important roles in regulating tissue-level radiation responses. CAFs contribute to extracellular matrix remodeling and secrete cytokines, chemokines, and growth factors that influence inflammation, fibrosis, tissue repair, and tumor progression [65,66]. Radiation exposure can alter CAF activation states and promote secretion of pro-fibrotic mediators, thereby reshaping ECM composition and intercellular signaling networks. These stromal alterations may subsequently influence epithelial cell behavior, establishing bidirectional interactions between stromal and epithelial compartments.

Emerging evidence further suggests that microenvironmental cues can drive divergent cellular outcomes from common progenitor populations [67,68]. In SVF-derived systems, cells exhibiting mesenchymal characteristics may undergo context-dependent transitions toward epithelial-like states under ECM-rich conditions, whereas alternative signaling environments may promote differentiation toward fibroblast- or CAF-like phenotypes [69,70]. This plasticity highlights the importance of environmental context in determining cell fate decisions and suggests that radiation-induced responses are shaped not only by intrinsic cellular properties but also by extrinsic microenvironmental signals [71].

The concept of fibrosis plasticity provides an important framework for integrating these observations [72]. Rather than representing a static endpoint, fibrosis may be viewed as a dynamic and potentially reversible process regulated by microenvironmental signaling networks and intercellular communication [73]. Radiation-induced fibrosis therefore reflects coordinated interactions among epithelial cells, fibroblasts, immune populations, and ECM components within tissue-specific contexts [74]. This perspective is consistent with emerging evidence suggesting that conserved regulatory programs underlie fibrotic responses across multiple organs [75].

Importantly, 3D organotypic systems enable investigation of these complex multicellular interactions in ways that are not readily achievable in traditional experimental models [76,77]. By incorporating multiple cell types and ECM components, these systems provide valuable platforms for examining how radiation-induced signals propagate across cellular compartments and influence tissue-level outcomes [78]. Such approaches are particularly relevant for understanding long-term consequences of radiation exposure, including fibrosis progression, tissue remodeling, therapeutic resistance, and tumor microenvironment evolution [79].

**Figure 4:**
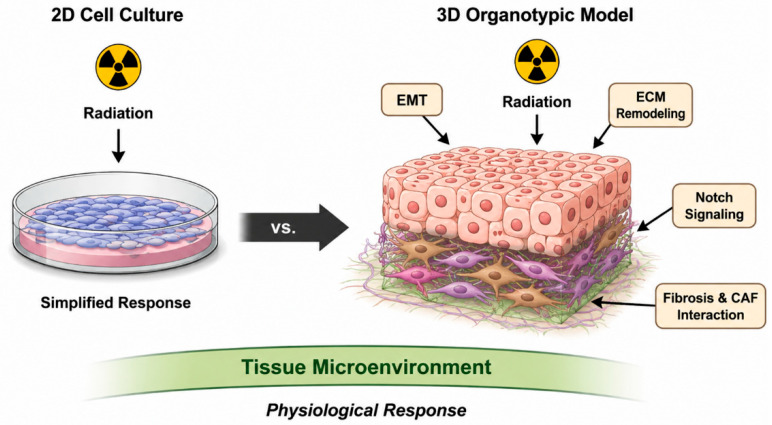
Comparison of radiation responses between conventional two-dimensional (2D) culture systems and three-dimensional (3D) organotypic models. Conventional 2D cell culture systems provide simplified and reductionist representations of radiation responses, primarily reflecting direct cellular effects in isolated monolayer conditions. In contrast, 3D organotypic models recapitulate key features of the tissue microenvironment, including extracellular matrix (ECM) architecture, stromal interactions, and multicellular organization. Radiation exposure within 3D systems induces more physiologically relevant responses involving epithelial–mesenchymal transition (EMT), ECM remodeling, Notch signaling activation, and fibrosis- or cancer-associated fibroblast (CAF)-related interactions. These microenvironment-dependent processes collectively influence cellular plasticity, inflammatory signaling, tissue remodeling, and long-term radiation responses, highlighting the importance of organotypic systems for predictive and translational radiobiology. Abbreviations: CAF, cancer-associated fibroblast; ECM, extracellular matrix; EMT, epithelial–mesenchymal transition. This figure was created and arranged by the author using Microsoft PowerPoint with manually assembled graphical elements and schematic illustrations.

Collectively, these findings demonstrate that the tissue microenvironment is not merely a passive structural background but an active regulator of radiation responses. Integration of 3D experimental systems with molecular, network-level, and systems biology analyses is therefore essential for capturing the full spectrum of radiation-induced biological effects. This organizational layer represents a critical component of the proposed multiscale framework, linking intracellular regulatory processes to tissue-level phenotypes and ultimately to organismal outcomes (Figure 4).

## 5. Non-Linear and Context-Dependent Radiation Responses

Radiation-induced biological responses are inherently non-linear and are strongly influenced by radiation dose, temporal dynamics, radiation quality, and biological context [80,81]. Although classical radiobiological models have traditionally assumed proportional relationships between radiation dose and biological effect, accumulating evidence indicates that such assumptions are insufficient to explain the diversity, heterogeneity, and context dependency of observed radiation responses. These limitations become particularly apparent under low-dose (<100 mGy), chronic, or fractionated exposure conditions, where adaptive responses, non-targeted effects, and regulatory network dynamics may substantially influence biological outcomes.

At low-dose ranges, radiation can induce diverse non-linear biological phenomena, including adaptive responses, low-dose hypersensitivity, and hormetic-like effects. Adaptive responses involve activation of protective mechanisms, such as enhanced DNA repair capacity, antioxidant defenses, immune modulation, and stress-response signaling, following prior low-dose exposure [82]. In contrast, low-dose hypersensitivity has been associated with insufficient activation of DNA repair or checkpoint pathways, resulting in disproportionately elevated biological effects relative to dose [83]. These observations suggest that low-dose radiation responses are shaped not only by absorbed dose but also by exposure history, radiation quality, dose rate, and cellular context.

Temporal dynamics further contribute to the non-linearity and complexity of radiation responses. Immediate molecular events, including DNA damage recognition, checkpoint activation, and repair signaling, occur within minutes to hours following irradiation. These early responses are subsequently followed by delayed and evolving processes, such as stress-responsive transcriptional reprogramming, epigenetic alterations, post-transcriptional regulation, and non-targeted signaling effects, which may persist over extended time scales [84,85]. Importantly, these delayed regulatory responses can substantially influence long-term biological outcomes, including fibrosis progression, tissue remodeling, chronic inflammation, therapeutic resistance, and carcinogenesis.

Biological heterogeneity introduces an additional layer of complexity into radiation responses. Cellular behaviors vary substantially according to cell type, differentiation state, metabolic activity, and tissue microenvironment [86]. For example, epithelial cells, fibroblasts, endothelial cells, and immune populations exhibit distinct radiation sensitivities and adaptive regulatory responses. Furthermore, studies using 3D organotypic systems have demonstrated that ECM composition, mechanical properties, and multicellular interactions can profoundly influence radiation-induced phenotypes. These observations suggest that radiation responses emerge from complex interactions across biological systems rather than from isolated linear molecular events alone.

From a systems biology perspective, non-linearity arises through feedback regulation, threshold effects, pathway cross-talk, and signal amplification across interconnected signaling networks [87]. Relatively subtle perturbations occurring at one regulatory level, such as altered RNA stability or cytokine signaling, may propagate through these networks and generate disproportionately large phenotypic consequences [88]. Such amplification may be particularly relevant under low-dose conditions, where modest molecular perturbations can progressively reshape long-term cellular and tissue phenotypes.

**Figure 5:**
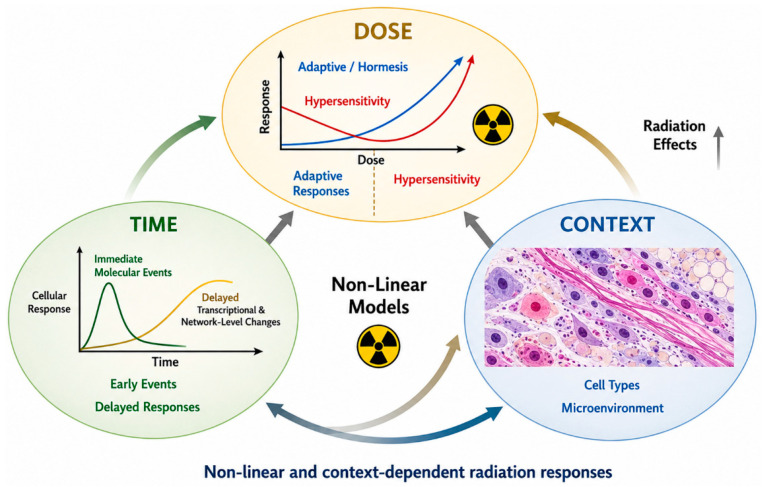
Non-linear and context-dependent radiation responses across biological scales. Radiation-induced biological effects are shaped by dynamic interactions among radiation dose, temporal progression, and biological context, resulting in non-linear and context-dependent responses. Dose-dependent effects may include adaptive responses, hormesis-like signaling, and hypersensitivity phenomena, particularly under low-dose exposure conditions. Temporal dynamics further influence radiation outcomes through immediate molecular responses followed by delayed transcriptional, signaling, and network-level alterations. In parallel, cellular composition and tissue microenvironmental conditions, including stromal interactions and intercellular communication, modulate the magnitude and direction of radiation responses. These interconnected factors collectively contribute to emergent biological behaviors that cannot be fully explained by linear dose–response models alone. The figure illustrates how dose, time, and biological context interact to influence radiation effects across molecular, cellular, and tissue levels within non-linear predictive radiobiology frameworks. This figure was created and arranged by the author using Microsoft PowerPoint with manually assembled graphical elements and schematic illustrations.

Collectively, these findings indicate that radiation responses are governed by multiscale interactions that are fundamentally non-linear and context dependent. This complexity presents a major challenge for prediction using traditional reductionist models while simultaneously emphasizing the need for integrative frameworks capable of incorporating genomic, regulatory, and microenvironmental information across biological scales. In this context, understanding non-linearity is not merely a descriptive objective but a prerequisite for developing predictive radiobiology models capable of improving radiation risk assessment, radiosensitivity prediction, and therapeutic response evaluation. These considerations further provide a conceptual foundation for computational and artificial intelligence (AI)-based approaches that are particularly well suited for modeling complex non-linear relationships across multiscale biological systems (Figure 5).

## 6. Artificial Intelligence for Predictive Radiobiology

Artificial intelligence (AI) and machine learning approaches are emerging as powerful computational frameworks for integrating multiscale biological data and modeling complex non-linear systems that are difficult to capture using conventional analytical methods [89]. In radiobiology, biological responses emerge through interactions across genomic damage, post-transcriptional regulation, signaling network dynamics, tissue microenvironmental interactions, and temporal adaptation processes. The complexity and heterogeneity of these interactions present substantial challenges for traditional reductionist modeling approaches and motivate the development of integrative computational strategies.

Machine learning methods, including supervised learning, unsupervised learning, and deep neural network-based approaches, have been increasingly applied to predict radiation sensitivity, therapeutic response, and normal tissue toxicity [90]. Importantly, these approaches enable integration of heterogeneous datasets, including genomic and transcriptomic profiles, multi-omics data, imaging information, spatial analyses, and clinical parameters. Such integrative modeling may facilitate identification of complex relationships across biological scales that are not readily detectable using conventional statistical frameworks.

AI-based approaches may be particularly valuable for modeling non-linear and context-dependent radiation responses, as discussed in previous sections. By identifying multidimensional interactions within complex datasets, AI models may capture relationships among DNA damage signatures, post-transcriptional regulation, inflammatory signaling, network rewiring, and tissue microenvironmental factors [91]. In this context, AI should not be viewed merely as a predictive tool, but as a systems-level framework for integrating interconnected biological processes across multiple organizational layers [92].

Recent advances in multi-omics technologies, spatial biology, and 3D organotypic systems provide increasingly rich experimental datasets that can serve as inputs for AI-based radiobiology models [93]. In particular, organotypic systems that recapitulate tissue-level complexity may generate experimentally controlled datasets suitable for investigating how molecular perturbations propagate into tissue-level phenotypes, including fibrosis progression, tumor microenvironment evolution, and therapeutic resistance [94]. Integration of these experimental systems with computational modeling may therefore improve both mechanistic understanding and predictive performance [95].

Despite these opportunities, several important challenges remain in the application of AI to radiobiology. Data heterogeneity, limited sample size, variability in experimental conditions, and lack of standardized datasets can substantially limit model robustness and generalizability [96]. Furthermore, many deep learning models operate as “black boxes,” making biological interpretation difficult and potentially limiting clinical applicability. These limitations are particularly important in radiobiology, where mechanistic interpretability is essential for understanding causal biological processes and improving translational relevance.

To address these issues, explainable artificial intelligence (XAI) approaches are increasingly being developed to improve model transparency and identify biologically meaningful features underlying prediction outcomes [96,97]. In parallel, integration of experimentally validated mechanistic information with AI-based modeling may help bridge the gap between computational prediction and biological interpretation [96,97]. Such approaches may ultimately facilitate the development of clinically relevant predictive frameworks capable of improving radiation risk assessment, radiosensitivity prediction, toxicity evaluation, and radiotherapy optimization [98].

**Figure 6:**
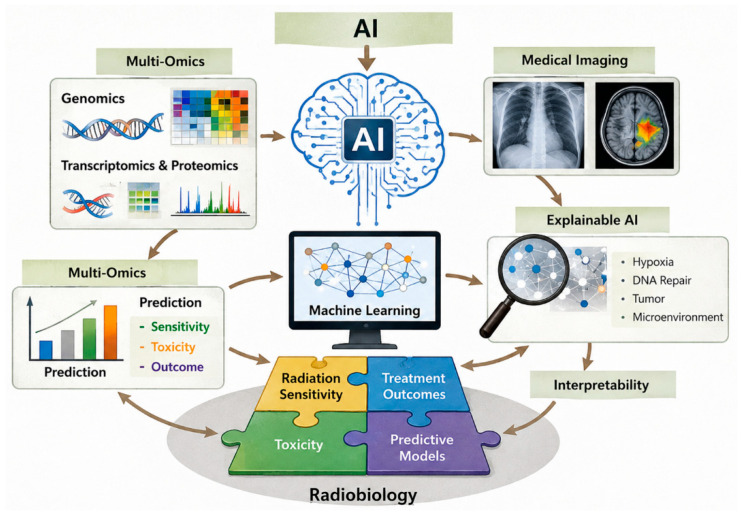
Artificial intelligence (AI)-based integration of multi-omics and imaging data for predictive radiobiology. Artificial intelligence (AI) and machine learning approaches provide computational frameworks for integrating heterogeneous biological and clinical datasets in predictive radiobiology. Multi-omics information, including genomic, transcriptomic, and proteomic datasets, together with medical imaging data, may be incorporated into AI-driven analytical pipelines to identify complex biological relationships associated with radiation responses. Machine learning models can facilitate prediction of radiosensitivity, treatment outcomes, radiation-induced toxicity, and patient-specific therapeutic responses. In addition, explainable AI approaches may improve interpretability of predictive models by identifying biologically relevant features associated with hypoxia, DNA repair capacity, and tumor microenvironmental characteristics. Integration of multi-scale biological information through AI-based frameworks may therefore support development of predictive models for individualized radiotherapy optimization and translational radiobiology. Abbreviations: AI, artificial intelligence. This figure was created and arranged by the author using Microsoft PowerPoint with manually assembled graphical elements and schematic illustrations.

Collectively, the integration of AI with multiscale experimental and biological data represents an important step toward predictive radiobiology. Rather than relying solely on descriptive or reductionist approaches, AI-assisted frameworks may facilitate identification of emergent properties, context-dependent behaviors, and cross-scale interactions that underlie complex radiation responses. These integrative strategies may ultimately support the transition from descriptive radiobiology toward more mechanistic and predictive models of radiation response (Figure 6).

## 7. Toward a Multiscale Predictive Framework

The integration of findings across genomic, post-transcriptional, network, and microenvironmental levels highlights the need for a unified multiscale framework in radiobiology [99]. Radiation-induced biological effects do not arise from isolated events occurring within a single biological layer. Rather, they emerge through dynamic interactions among interconnected molecular, cellular, tissue, and temporal processes that collectively shape context-dependent phenotypic outcomes.

Within this framework, DNA damage represents the initial perturbation that activates downstream regulatory responses. However, the ultimate biological consequences of radiation exposure are shaped by how these perturbations are propagated, integrated, and modified across multiple regulatory layers [100]. Post-transcriptional regulation mediated by RNA-binding proteins, signaling network rewiring, inflammatory communication, and tissue microenvironmental interactions all contribute to shaping cellular adaptation and long-term tissue responses [100,101].

Importantly, these biological layers do not function independently. Instead, perturbations occurring at one level may be amplified, buffered, or redirected through interactions occurring at other organizational scales [102]. For example, DNA damage-induced signaling may alter RNA stability and cytokine production, which subsequently influence intercellular communication, stromal activation, extracellular matrix remodeling, and tissue-level phenotypes [103]. Conversely, microenvironmental conditions may modulate DNA repair efficiency, signaling dynamics, and cellular stress responses [66]. These bidirectional interactions suggest that radiation responses are fundamentally emergent properties of multiscale biological systems [102].

The relative contribution of each regulatory layer may further vary according to radiation dose, radiation quality, temporal progression, and tissue context [102]. Under high-dose or high-LET exposure conditions, direct genomic damage and chromosomal instability may dominate biological outcomes [104]. In contrast, under low-dose or chronic exposure conditions, post-transcriptional regulation, inflammatory signaling, and adaptive network-level responses may exert proportionally greater influence despite relatively limited detectable genomic damage [34,105]. These observations further emphasize the limitations of reductionist frameworks based solely on DNA lesion accumulation [102].

From a systems biology perspective, predictive radiobiology therefore requires integration of heterogeneous biological information across multiple scales, including genomic damage signatures, RNA regulatory dynamics, signaling network behavior, cellular plasticity, and tissue microenvironmental interactions. Within this conceptual framework, computational approaches, including artificial intelligence (AI) and network-based modeling, serve as important tools for integrating high-dimensional datasets and identifying complex relationships that may underlie radiation responses [106,107]. Importantly, AI should be viewed not as a replacement for mechanistic radiobiology, but as a complementary framework for integrating multiscale biological information and improving predictive capability.

The multiscale framework proposed here is intended not merely as a descriptive model, but as a foundation for predictive and translational radiobiology [108]. By explicitly incorporating interactions across biological layers, this framework may facilitate identification of critical determinants of radiosensitivity, tissue toxicity, fibrosis progression, therapeutic resistance, and long-term carcinogenic risk [109]. Such integrative approaches may ultimately contribute to radiotherapy optimization, individualized radiation risk assessment, biomarker discovery, and the development of mitigation strategies for radiation-induced tissue damage [110].

**Figure 7:**
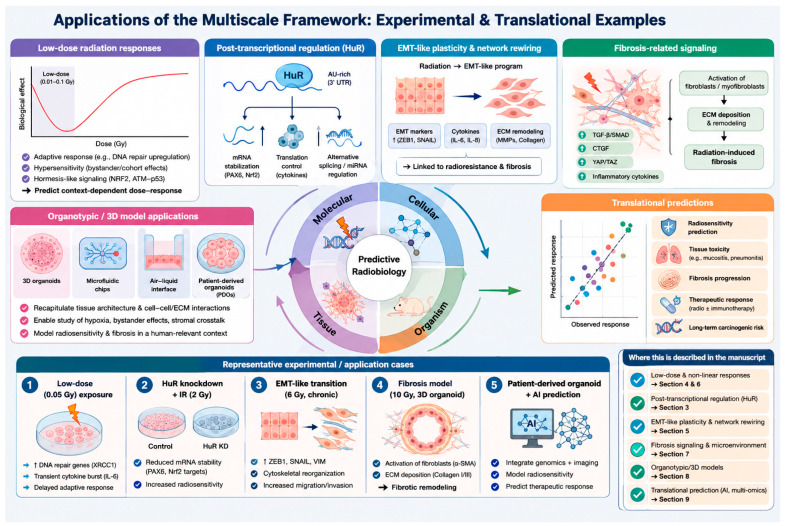
Representative experimental and translational applications of the multiscale predictive radiobiology framework. This figure summarizes representative biological contexts, experimental systems, and translational applications illustrating how the proposed multiscale framework may be applied to predictive radiobiology. Low-dose radiation responses involve non-linear biological effects, including adaptive responses, hypersensitivity, and hormesis-like signaling. Post-transcriptional regulation mediated by RNA-binding proteins such as HuR contributes to modulation of mRNA stability, translation, and inflammatory signaling under radiation stress conditions. Radiation-induced EMT-like plasticity and network rewiring promote cellular adaptation, extracellular matrix (ECM) remodeling, and fibrosis-associated phenotypes through cytokine signaling and microenvironmental interactions. Fibrosis-related signaling pathways, including TGF-β/SMAD, CTGF, and YAP/TAZ, contribute to fibroblast activation and radiation-induced tissue remodeling. In parallel, organotypic and three-dimensional (3D) experimental systems, including organoids, organ-on-chip platforms, and patient-derived organoids, provide physiologically relevant models for investigating radiation responses within complex tissue microenvironments. Integration of these experimental platforms with multi-omics datasets and artificial intelligence (AI)-based computational approaches may facilitate prediction of radiosensitivity, tissue toxicity, fibrosis progression, therapeutic response, and long-term carcinogenic risk across multiple biological scales. Abbreviations: AI, artificial intelligence; ATM, ataxia telangiectasia mutated; CTGF, connective tissue growth factor; ECM, extracellular matrix; EMT, epithelial–mesenchymal transition; HuR, human antigen R; IR, ionizing radiation; KD, knockdown; NRF2, nuclear factor erythroid 2–related factor 2; PDOs, patient-derived organoids; SMAD, suppressor of mothers against decapentaplegic; TGF-β, transforming growth factor-β; YAP/TAZ, yes-associated protein/transcriptional coactivator with PDZ-binding motif. This figure was created and arranged by the author using Microsoft PowerPoint with manually assembled graphical elements and schematic illustrations.

Taken together, these perspectives support a transition from reductionist and predominantly DNA-centric models toward integrative predictive radiobiology frameworks capable of capturing the complexity, heterogeneity, and context dependence of radiation responses across biological scales (Figure 7).

## 8. Limitations and Challenges in Multiscale Predictive Radiobiology

Despite recent advances in multi-omics technologies, systems biology, and organotypic modeling, several conceptual, technical, and translational challenges remain in the development of predictive multiscale radiobiology frameworks [111]. One of the major challenges is the substantial heterogeneity of biological datasets generated across different experimental systems, radiation qualities, dose ranges, and analytical platforms [111]. Variability in sample preparation, sequencing methodologies, computational pipelines, and experimental conditions can significantly affect reproducibility and limit cross-study comparisons [111,112].

A further challenge involves the integration of heterogeneous data across multiple biological scales. Although genomic, transcriptomic, proteomic, spatial, and imaging datasets each provide valuable information, combining these datasets into unified predictive models remains technically and conceptually challenging [113]. Biological responses to radiation are highly dynamic and context-dependent, and the relative contribution of individual regulatory layers may vary according to radiation dose, temporal progression, tissue type, and microenvironmental conditions [93]. These factors complicate the identification of universally applicable predictive biomarkers or modeling strategies.

In addition, although three-dimensional (3D) organotypic systems and organoids provide improved physiological relevance compared with conventional two-dimensional culture systems, these models still possess important limitations [114]. Many current organoid systems incompletely recapitulate vascular, immune, and systemic interactions present in vivo [115]. Furthermore, long-term tissue remodeling, organism-level adaptation, and inter-organ communication remain difficult to model experimentally [116]. Consequently, findings obtained from in vitro or ex vivo systems may not fully translate to clinical radiation responses.

The application of artificial intelligence (AI) and machine learning approaches to radiobiology also presents several challenges [117,118]. Many AI models require large, high-quality datasets for robust training and validation, whereas radiobiological datasets are frequently limited by small sample sizes and experimental variability [119]. In addition, deep learning approaches often function as “black boxes,” making mechanistic interpretation difficult and potentially limiting mechanistic interpretability and clinical confidence [117,118]. Although explainable AI (XAI) approaches are increasingly being developed, achieving an appropriate balance between predictive performance and interpretability remains an ongoing challenge [117,118].

An additional challenge is the relative scarcity of longitudinal human datasets integrating molecular, tissue-level, and clinical outcome information following radiation exposure [95,120,121]. Most current studies remain focused on isolated biological layers or short-term experimental endpoints [120,121]. Future progress in predictive radiobiology will likely require standardized multi-institutional datasets, longitudinal clinical validation, and closer integration between experimental radiobiology, computational modeling, and translational research [120,121].

Finally, it is important to recognize that the multiscale framework proposed in this review is intended primarily as a conceptual and integrative perspective rather than a finalized predictive model [95,102]. Although this framework may help organize emerging knowledge across multiple biological layers, substantial work remains necessary to determine which regulatory interactions are most critical for specific radiation outcomes and how these interactions can be quantitatively modeled in clinically relevant contexts [117].

Collectively, these limitations highlight both the complexity and the opportunity of predictive radiobiology. Addressing these challenges will require interdisciplinary collaboration across radiobiology, systems biology, computational modeling, bioinformatics, and clinical radiation sciences to develop more robust, interpretable, and biologically interpretable and translationally relevant predictive frameworks.

## 9. Conclusions and Future Perspectives

Radiobiology is increasingly transitioning from predominantly DNA-centric and linear models toward more integrative and multiscale perspectives of radiation response. Although DNA damage remains a critical initiating event, accumulating evidence indicates that radiation-induced biological outcomes are governed by complex interactions among genomic damage, post-transcriptional regulation, signaling network dynamics, and tissue microenvironmental context. These interconnected processes collectively shape the heterogeneity, non-linearity, and context dependence of radiation responses across biological scales.

Advances in multi-omics technologies, spatial biology, systems biology, and 3D organotypic modeling have substantially expanded the ability to investigate these multiscale processes in physiologically relevant contexts. In particular, emerging evidence suggests that post-transcriptional regulation and network rewiring may play important roles in mediating radiation responses under conditions where detectable genomic damage alone is insufficient to explain observed biological effects, including low-dose and chronic radiation exposure scenarios.

The incorporation of tissue microenvironmental context through 3D experimental systems further emphasizes that radiation responses are not determined solely by intrinsic cellular properties, but also by interactions among epithelial, stromal, immune, and extracellular matrix components. These findings support the concept that radiation-induced phenotypes represent emergent properties of interconnected biological systems rather than isolated molecular events.

Importantly, the complexity and non-linearity of radiation responses present major challenges for conventional reductionist prediction models. In this context, computational approaches, including artificial intelligence (AI) and systems-level modeling, may provide valuable tools for integrating heterogeneous datasets and identifying cross-scale regulatory relationships. However, substantial challenges remain, including data heterogeneity, limited reproducibility across experimental systems, insufficient standardization of multi-omics datasets, and the need for improved mechanistic interpretability and clinically relevant validation models.

The multiscale framework proposed in this review provides a conceptual foundation for predictive radiobiology by integrating molecular, regulatory, network-level, and tissue-scale processes into a unified perspective of radiation response. Such integrative approaches may facilitate improved prediction of radiosensitivity, normal tissue toxicity, fibrosis progression, therapeutic resistance, and long-term carcinogenic risk. Furthermore, these frameworks may contribute to radiotherapy optimization, individualized risk assessment, biomarker discovery, and development of mitigation strategies for radiation-induced tissue damage.

Future studies integrating multi-omics profiling, spatially resolved analyses, organotypic systems, and interpretable AI-based computational approaches will likely be essential for advancing predictive and translational radiobiology. Collectively, these efforts support a broader transition from descriptive radiobiology toward mechanistically informed and context-aware predictive models capable of capturing the complexity of radiation-induced biological responses.

## Data Availability

No new data were created or analyzed in this study. Data sharing is not applicable to this article.
